# Volatile signalling by sesquiterpenes from ectomycorrhizal fungi reprogrammes root architecture

**DOI:** 10.1038/ncomms7279

**Published:** 2015-02-23

**Authors:** Franck A. Ditengou, Anna Müller, Maaria Rosenkranz, Judith Felten, Hanna Lasok, Maja Miloradovic van Doorn, Valerie Legué, Klaus Palme, Jörg-Peter Schnitzler, Andrea Polle

**Affiliations:** 1 Institute of Biology II, Faculty of Biology, Albert-Ludwigs-University of Freiburg, Schänzlestrasse 1, D-79104 Freiburg, Germany; 2 Forest Botany and Tree Physiology, Georg-August Universität Göttingen, Büsgenweg 2, D-37077 Göttingen, Germany; 3 Research Unit Environmental Simulation, Institute of Biochemical Plant Pathology, Helmholtz Zentrum München—German Research Center for Environmental Health (GmbH), Ingolstädter Landstrasse 1, D-85764 Neuherberg, Germany; 4 Umeå Plant Science Center, Department for Forest Genetics and Plant Physiology, Swedish University of Agricultural Sciences, SE-901 83 Umeå, Sweden; 5 INRA and Lorraine University, UMR 1136, Interactions Arbres/Micro-organismes, Centre INRA de Nancy, 54280 Champenoux, France; 6 BIOSS Centre of Biological Systems Analysis, Albert-Ludwigs-University of Freiburg, Habsburgerstrasse 49, D-79104 Freiburg, Germany; 7 Freiburg Institute of Advanced Sciences (FRIAS), Albert-Ludwigs-University of Freiburg, Albertstrasse 19, D-79104 Freiburg, Germany; 8 Centre for Biological Signalling Studies (BIOSS), Albert-Ludwigs-University of Freiburg, Schänzlestrasse 18, D-79104 Freiburg, Germany

## Abstract

The mutualistic association of roots with ectomycorrhizal fungi promotes plant health and is a hallmark of boreal and temperate forests worldwide. In the pre-colonization phase, before direct contact, lateral root (LR) production is massively stimulated, yet little is known about the signals exchanged during this step. Here, we identify sesquiterpenes (SQTs) as biologically active agents emitted by *Laccaria bicolor* while interacting with *Populus* or *Arabidopsis.* We show that inhibition of fungal SQT production by lovastatin strongly reduces LR proliferation and that (–)-thujopsene, a low-abundance SQT, is sufficient to stimulate LR formation in the absence of the fungus. Further, we show that the ectomycorrhizal ascomycote, *Cenococcum geophilum*, which cannot synthesize SQTs, does not promote LRs. We propose that the LR-promoting SQT signal creates a win-win situation by enhancing the root surface area for plant nutrient uptake and by improving fungal access to plant-derived carbon via root exudates.

## Abstract (web summary)

Soil-dwelling ectomycorrhizal fungi trigger remodelling of root architecture as part of a mutualistic symbiotic relationship with host plants. Here, Ditengou *et al.* identify fungal-derived sesquiterpenes as volatile signals capable of stimulating lateral root production in *Arabidopsis* and *Populus*.

## Graphical abstract

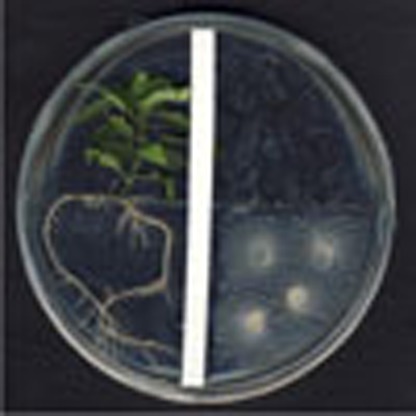

Most land plants are associated with mycorrhizal fungi that help them to maintain water and nutrient supply under environmental constraints. The interactions between tree roots and mutualistic, ectomycorrhizal soil fungi can lead to profound modifications of root development, such as a massive stimulation of lateral root (LR) formation^1^.

LR stimulation by fungi is independent of root colonization and functional symbiosis, because non-host plants such as *Arabidopsis thaliana* also develop more LRs in contact with *Laccaria bicolor*, a typical ectomycorrhizal fungus^2^. Because enhanced LR production is observed even without physical contact, when a membrane permeable to small solutes separates the two partners, stimulation of LR development has been ascribed to diffusible fungal signals^2^.

Little is known about the nature of the signals exchanged between fungi and plants. Phytohormones, such as auxin and the gaseous ethylene presumably released by mycorrhizal fungi, have been suggested to play central roles as signal molecules in fungal–plant interactions^2^,^3^. Although the application of these compounds could mimic fungal effects, it is unknown whether phytohormones of fungal origin are the prime trigger for LR proliferation *in situ* because of divergent timing of auxin- and fungal-induced LR development and decreased auxin levels in ectomycorrhizal root interactions^3^,^4^. Therefore other molecules may serve as the primary signal. It has been suggested that volatile organic compounds (VOCs) could be involved in LR induction.

Fungi emit a broad spectrum of VOCs with diverse ecological functions^5^,^6^. Fungal VOCs can exhibit antibiotic effects^6^,^7^,^8^ and inhibit plant growth^9^. However, growth-promoting effects on plants by VOCs released from pathogenic or saprotrophic soil fungi have also been reported^10^,^11^,^12^. Ectomycorrhizal fungi emit complex, species-specific odour profiles, which distinguish them from fungi of other ecological niches^13^. Whether ectomycorrhizal VOCs are required for the interaction with plant roots, is currently unknown.

In the present study, we investigated whether VOCs from ectomycorrhizal fungi participate in the early communication with plant roots. We characterized the VOC patterns of plant roots and ectomycorrhizal fungi without direct physical interaction. We demonstrated that sesquiterpenes (SQTs) emitted by *L. bicolor* are instrumental for inducing LR growth in the host plant *Populus* and the non-mycorrhizal plant *Arabidopsis*. The ectomycorrhizal ascomycote *Cenococcum geophilum*, which cannot synthesize SQTs, does not promote LRs when confronted with *Arabidopsis*, supporting the importance of SQTs as the main regulators of LR development during early *Laccaria*–plant root interaction. Among the VOCs released by *L*. *bicolor*, we identified the SQT (–)-thujopsene as a volatile signal sufficient to stimulate LR development. Moreover, we demonstrated that the downstream processing of the volatile signal also increased the root hair length and involved superoxide anion radical (O_2_^−^) formation in the meristematic zone of root tips.

## Results

### VOCs released by *Laccaria* promote LR growth

**Figure 1:**
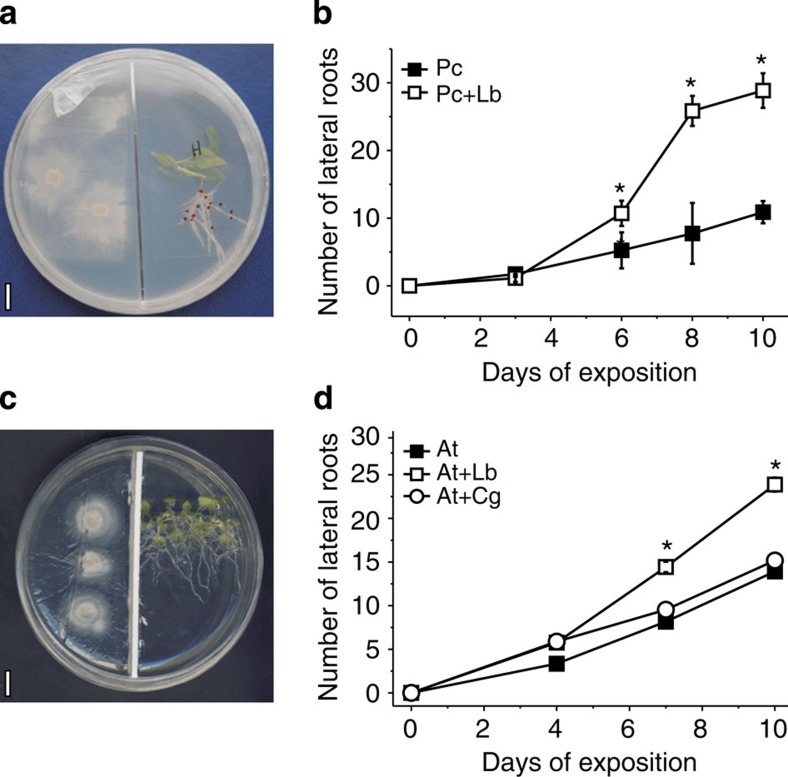
*Laccaria* volatile organic compounds (VOCs) stimulate lateral root development in poplar and *Arabidopsis*. (**a**) *P. tremula* × *P. alba* (Pc) plantlets grown in the presence of *L. bicolor* (Lb) in a bi-compartmented Petri dish avoiding direct contact and solute exchange between the plant and the fungus. Red dots indicate lateral roots. Scale bar, 10 mm. (**b**) Time course of lateral root development. The poplar roots were exposed to fungal VOCs. (**c**) *Arabidopsis* seedlings (At) grown in the presence of *L bicolor* in a bi-compartmented Petri dish. Scale bar, 10 mm. (**d**) Time courses of lateral root development of *A. thaliana* grown in the presence of *L*. *bicolor* (Lb) or *C*. *geophilum* (Cg) VOCs. Asterisks (*) in **b** and **d** indicate significant differences compared with controls at *P*<0.05 (*t*-test); when error bars are not visible they were smaller than the symbols. Data are means (*n*=8–10, ±s.e.).

Poplar or *Arabidopsis* plants were grown in the presence of fungal VOCs by cultivating plants and *L. bicolor* mycelia in bi-compartmented Petri dishes (Fig. 1a–d), hereafter referred to as headspace co-cultivation. Despite the absence of direct contact with *L. bicolor*, strong stimulation of LR development was observed in both plant species, which must have been caused by volatile compounds (Fig. 1a–d). The stimulation of LR formation started after 4 d.h.c.c. (days of headspace co-cultivation) and resulted in almost twice the number of LR tips after 10 d.h.c.c. with *L. bicolor* (Fig. 1b,d). In closed growth systems, fluctuations in CO_2_ availability due to plant CO_2_ consumption and release by fungal respiration can influence LR formation^14^. We excluded the possibility that LR stimulation under headspace co-cultivation conditions was solely an effect of altered CO_2_ levels caused by the presence of the mycelium (Supplementary Fig. 1a–c). Therefore, besides CO_2_, other VOCs (OVOCs) must be involved in LR induction. *C. geophilum*, a mycorrhizal fungus known to emit only few different VOCs^13^, did not induce LR development (Fig. 1d). Because *C. geophilum* specifically lacks SQTs, a class of compounds previously shown to be biologically active^10^,^15^ and to diffuse well in the soil environment^16^, we suspected that this VOC class could be involved in LR formation.

*L. bicolor* VOCs promoted the growth of *Arabidopsis* primary and LRs (sum of the lengths of all LRs^17^) by 21 and 158% in comparison with control roots and control LRs, respectively (Supplementary Fig. 2a,b). To exclude that LR formation was only a function of the length of the main root, we calculated the LR density (number of LRs per root length). LR density increased by 27% in the presence of *L. bicolor* VOCs (Supplementary Fig. 2c). This result suggested that the fungus may also have promoted the initiation of LR primordia (LRP) (Supplementary Fig. 2c). To test this hypothesis, we quantified the number of LRs initiated along the portion of the root developed after 3 days and 7 days of headspace co-cultivation using DR5::GFP reporter fusion, a well-established marker of LRP^18^ (Supplementary Fig. 2d–g). *L. bicolor* VOC-treated plants displayed a higher density of LR per cm (0.5 after 3 days and 0.8 after 7 days) compared with non-treated plants (0.05 after 3 days and 0.1 after 7days) (Supplementary Fig. 2d). All together, these data demonstrate that *L*. *bicolor* VOCs promoted the initiation of LRP and their further elongation.

### *Laccaria* VOCs induce superoxide anion radicals in roots

**Figure 2:**
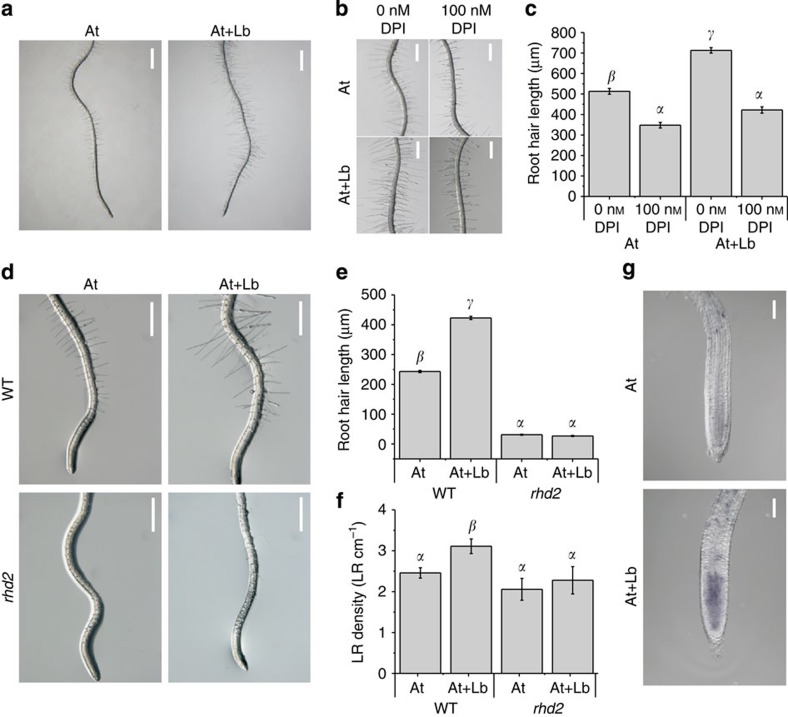
*L. bicolor* VOCs stimulate root hair growth through a ROS-dependent mechanism. (**a**) Root hairs of *Arabidopsis* plants (At) grown in the presence or absence of *L. bicolor* VOCs (Lb). Scale bar, 1,000 μm. (**b**) Diphenyleneiodium (100 nM, DPI) suppresses the *L. bicolor* VOC-mediated stimulation of root hairs. Scale bar=500 μm. (**c**) Quantification of root hair size of plants shown in **a**. Different letters indicate significant differences of the mean values at *P*<0.05 (HSD test, *n*=100–121). (**d**) Root hairs of Arabidopsis WT plants (At) and *rhd2* mutants grown in plates (pH 5.0) in the presence or absence of *L. bicolor* VOCs (Lb). Scale bar=500 μm. (**e**) Quantification of root hair size of plants shown in **d**. Different letters indicate significant differences of the mean values at *P*<0.05 (honest significant difference (HSD) test). (**f**) Quantification of LR density of plants shown in **d**. Different letters indicate significant differences of the mean values at *P*<0.05 (HSD test). (**g**) Superoxide anion radical staining reveals ROS accumulation in the root of an *Arabidopsis* plant grown in the presence of *L. bicolor* VOCs. Scale bar, 100 μm.

In addition to LR development, *L*. *bicolor* VOCs also promoted root hair growth (Fig. 2a). The root hairs of *L*. *bicolor* VOC-treated plants were on average 713±13 μm long versus only 513±14 μm for controls (mean±s.e., *n*=100–121, *P*=6.8 × 10^−18^ Student’s *t*-test). Overall, we observed a 39% increase in the root hair length under fungal VOC exposure (Fig. 2b). Because the polar tip growth of pollen tubes and root hairs is associated with reactive oxygen species (ROS) accumulation^19^, we tested whether the *L. bicolor* VOC-mediated stimulation of root hair growth was ROS dependent. In the presence of 100 nM diphenyleneiodium (DPI) (an inhibitor of nicotinamide adenine dinucleotide phosphate (NADPH) oxidase, an enzyme involved in O_2_^−.^ production^20^), the root hair length was similarly reduced in mock-treated and plants incubated with *L. bicolor* VOCs (Fig. 2b,c). The root hair length was reduced by 32% in control plants, and the presence of the fungus did not overcome this inhibition, suggesting that *L. bicolor* VOCs promote root hair growth through a ROS-dependent mechanism. A role of O_2_^−.^ in *L. bicolor* VOC-promoted root hair and LR formation was further tested in *Arabidopsis ROOT HAIR DEFECTIVE (RHD2)*-mutants. The *RHD2* gene encodes an NADPH oxidase that transfers electrons from NADPH to an electron acceptor, leading to the formation of ROS^19^. Because O_2_^−.^radicals are necessary for root hair growth, *rhd2* plants develop very short root hairs^19^. When grown with *L. bicolor* in the same headspace co-cultivation system, *rhd2* mutants were unresponsive to both root hair growth and LR stimulation by *L*. *bicolor* VOCs (Fig. 2d–f). These data suggest that RHD2-dependent O_2_^−.^production contributes to LR and root hair growth stimulation by *L. bicolor*. We then questioned whether *L. bicolor* VOCs could induce ROS accumulation in *Arabidopsis* roots. Superoxide anion radicals were detected by staining roots with nitroblue tetrazolium (NBT). Indeed, compared with control plants, 70% (*n*=15) of roots exposed to *L. bicolor* VOCs displayed stronger O_2_^−^ staining in the root tip than the control plants (Fig. 2g).

### *Laccaria* VOCs do not alter auxin signalling in roots

The phytohormone auxin controls several aspects of plant development, including LR development^21^. In earlier studies, an increase in the auxin response was visualized with the auxin-sensitive DR5::GFP reporter fusion in *Arabidopsis* root tips growing in contact with *L*. *bicolor*^2^. Here, we tested whether the influence of *L*. *bicolor* VOCs on root development also involved changes in auxin signalling in the root. Five-day-old *Arabidopsis* DR5::GFP plants were grown in headspace co-cultivation with *L. bicolor*, and the green fluorescent protein (GFP) signal was monitored after 3 and 5 d.h.c.c. We did neither observe a change in the auxin signal in any of the root tips nor along the root system, where LRs emerge, for plants cultivated in the presence of *L*. *bicolor* VOCs (Supplementary Fig. 2d–h). This observation suggests that the activity of VOCs on LR and root hair development does not involve changes in auxin signalling pathways.

### Identification of VOC patterns in plant–fungal co-cultures

To identify the VOCs responsible for LR induction, VOC patterns of *L. bicolor*, *C. geophilum*, *Arabidopsis* and plant–fungus combinations were measured in the headspace, and the released compounds were classified as monoterpenes (MTs), SQTs or OVOCs (Table 1; Supplementary Tables 1 and 2). Compared with other fungal species, *L. bicolor* and *C. geophilum* are weak VOC emitters with distinct differences in their emission patterns^15^. The most obvious was the difference in SQT abundance. The VOC profile of *L. bicolor* contained predominantly SQTs, whereas these were not observed in the presence of *C. geophilum* (Table 1).

*Arabidopsis* without fungal mycelium did not release any SQTs. The headspace co-culture of *Arabidopsis* and *L. bicolor* resulted in VOC patterns that differed slightly from those of the separately grown organisms. We observed a new SQT, valencene, in the co-culture, whereas two VOCs found in separate cultures of *Arabidopsis* or *L. bicolor*, were absent. Eleven SQTs that were identified in the headspace of separately grown *L. bicolor* were also present in the co-culture (Table 1). In the headspace co-cultures of *C. geophilum* and *Arabidopsis* no SQT and only one MT was observed.

### Sesquiterpenes are the main signal for the stimulation of LR

The presence of SQTs in the VOC profile of *L. bicolor* is likely to be important for LR stimulation, as their absence in *C. geophilum* correlates with the absence of LR induction. To test this hypothesis, we inhibited SQT biosynthesis in *L. bicolor* and studied the effect on LR stimulation in *Arabidopsis*. Lovastatin is a competitive inhibitor of the 3-hydroxy-3-methylglutaryl-CoA reductase, a key enzyme in the mevalonate pathway required for cytosolic isoprenoid and hence SQT biosynthesis^22^. Growth of *L. bicolor* on lovastatin-supplemented medium suppressed SQT biosynthesis (Table 1) but did not impair fungal growth. In the headspace co-cultivation system, we added lovastatin only in the fungal compartment to prevent any direct effects of the inhibitor on the plant. Thereby, the overall SQT abundance released by the fungus into the headspace of 10-day-old co-cultures was significantly reduced from 14.8±3.4 to 6.1±2.2 pmol cm^−2^ SQTs per mycelium surface area (mean±s.e., *n*=10 plates per treatment, *P*=0.024, Student’s *t*-test). The suppressed SQT biosynthesis resulted in the disappearance of five SQTs: β-caryophyllene, (–)-thujopsene, γ-cadinene, isoledene and cadina-1,4-diene (Table 1). In contrast to the SQTs, the amounts of MTs and OVOCs were not significantly affected by lovastatin treatment (Table 1).

**Figure 3:**
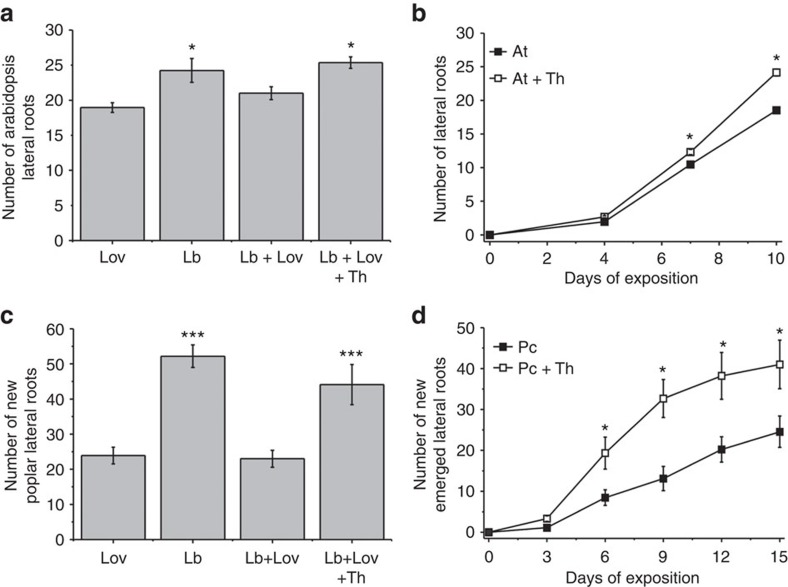
Sesquiterpenes (SQTs) are the main regulators of LR development. (**a**) In the presence of Lovastin (Lov), an inhibitor of SQT synthesis, the *L. bicolor* (Lb)-induced lateral root formation of *Arabidopsis* (At) is abolished. *Arabidopsis* seedlings were grown in bi-compartmented Petri dishes without physical contact to the second compartment, which was supplemented with *L. bicolor* (Lb) in the presence or absence of Lov (5 μM). The SQT (−)-thujopsene (Th, 100 p.p.b.) rescues lateral root formation when *L. bicolor* SQT production was inhibited by Lov (Lb+Lov+Th). Plants were analysed after 10 days of exposure. Controls in the absence of Lov are shown in **b**. The asterisks indicate significant differences (Tukey’s test, *P*<0.05; *n*=10, mean±s.e.). (**b**) The SQT (−)-thujopsene (Th) induces LR formation of *Arabidopsis* seedlings. In bi-compartmented Petri dishes, the plants were grown on one side and a filter paper with the VOC (100 p.p.b.) was placed on the other side. The asterisks (*) indicate significant differences to mock-treated *Arabidopsis* controls (Student’s *t-*test, *P*<0.05; *n*=10, mean±s.e.). (**c**) In the presence of Lov, the *L. bicolor* (Lb)-induced lateral root formation of poplar plantlets (Pc) is abolished. Poplar plantlets were grown in bi-compartmented Petri dishes without physical contact to the second compartment, which was supplemented *L. bicolor* (Lb) in the presence or absence of Lov (5 μM). The SQT (−)-thujopsene (Th, 100 p.p.b.) rescues lateral root formation when *L. bicolor* SQT production was inhibited by Lov (Lb+Lov+Th). Plants were analysed after 10 days of exposure. Controls in the absence of Lov are shown in **d**. The asterisks (***) indicate significant differences (Tukey’s test, *P*<0.001; *n*=8–10, mean±s.e.). (**d**) (−)-Thujopsene (Th) induces LR formation in poplar plantlets (Pc), which were grown in bi-compartmented Petri dishes with (−)-thujopsene (100 p.p.b.) on filter paper in the adjacent compartment. The asterisks (*) indicate significant differences to mock-treated control poplars (Student’s *t-*test, *P*<0.05; *n*=9; mean±s.e.).

*Arabidopsis* and poplar plants developing their root system under the altered VOC profile, in which distinct fungal SQTs were suppressed by lovastatin (Table 1), did not show stimulation of LR formation (Fig. 3a,c; Supplementary Fig. 3a). These results strongly support a role of SQTs in VOC-induced LR formation.

Because the two SQTs (–)-thujopsene and β-caryophyllene were most strongly suppressed by lovastatin treatment, we investigated their involvement in LR formation. Both SQTs were tested independently at increasing concentrations during headspace co-cultivation for their ability to induce LR (Supplementary Fig. 3b–f). β-Caryophyllene had no effect on LR formation, neither in *Arabidopsis* nor in poplar (Supplementary Fig. 3d,f). (–)-Thujopsene, employed at a concentration corresponding to 6.4 pmol after 6 h of headspace collection, stimulated LR formation in *Arabidopsis* by ~30% (Fig. 3b). The influence of (–)-thujopsene on poplar was even stronger than on *Arabidopsis*, where approximately twice as many LRs were observed in the presence than in the absence of this SQT (Fig. 3d). When fungal SQT biosynthesis was blocked by lovastatin, the addition of (–)-thujopsene to the headspace fully rescued LR stimulation in both *Arabidopsis* and poplar (Fig. 3a,c).

## Discussion

The establishment of the ectomycorrhizal symbiosis requires the exchange of signals between the plant and its fungal partner^23^,^24^,^25^,^26^,^27^. Chemical messengers are necessary for both partners to recognize each other. Little is known about the nature of the molecules that regulate the early phases of the interaction between ectomycorrhizal fungi and their hosts. Recent studies with *L. bicolor* and truffle (*Tuber borchii*) have demonstrated that the initial phase of the symbiosis, prior to root colonization by the fungus, is associated with a massive LR stimulation^2^,^3^,^28^. Because sealed Petri dishes were used in those studies, the growth-promoting effects could be due to an increase in the CO_2_ concentration due to fungal respiration^14^. Our data show that *L. bicolor*-induced LR stimulation did not disappear when the CO_2_ levels in the headspace co-cultivation were diminished, hence, demonstrating that the increased LR formation by *L. bicolor* was not due to fungal CO_2_ release. Several studies have suggested roles for auxin and ethylene in ectomycorrhiza-induced root branching^3^,^29^,^30^. While auxin produced by mycorrhizal fungi^3^,^31^ requires a relatively close contact with the plant partner to make an impact, our experiments clearly indicate that the exchange of molecules between the ectomycorrhizal fungus and the plant via the aqueous phase is not necessary for LR stimulation. Our results stress the role of fungal VOCs as signalling molecules in the early phase of LR formation. Moreover, we identified SQTs as the biologically active agents for this interaction. Whether and how ethylene, another volatile compound with functions in early plastic adaptation of roots to external stimuli^3^,^32^, integrates into these processes needs to be analysed in future studies. Our study clearly demonstrates that *L*. *bicolor* VOCs can act as volatile signals on a typical host (*Populus* × *canescen*s) and on a non-host plant species (*Arabidopsis thaliana*).

Because LRs^2^,^3^ and root hair^3^ development were reported to be associated with changed auxin responses in *Arabidopsis* roots in close proximity with *L. bicolor*^2^ or *T. borchii*^3^ mycelia, we tested whether auxin distribution was affected in the presence of *L. bicolor* odours. We did not observe any change in the auxin-sensitive DR5::GFP signal intensity or spatial distribution in our experimental conditions, suggesting that auxin signalling is not the first target of *L*. *bicolor* VOCs. This result stresses the possibility that *L*. *bicolor* VOCs may activate other signalling routes in the plant.

ROS are secondary messengers that mediate the developmental reprogramming of roots^33^,^34^,^35^,^36^, and are key players in triggering and modulating stress responses^37^,^38^. Early after mycorrhizal infection, waves of ROS production have been observed^39^, and mature mycorrhizas accumulate H_2_O_2_ in the hyphal mantle, ensheathing the root tip^40^. ROS are therefore thought to contribute to mycorrhizal priming and protect roots against environmental cues^4^,^41^. O_2_^−.^production, through the activity of the NADPH oxidase, is also critical for root hair growth in *Arabidopsis*^19^, as well as for LR development in *Phaseolus vulgaris*^42^,^43^. Our data demonstrate that *L*. *bicolor* VOCs promoted root hair growth and LR development, two processes that are sensitive to O_2_^−^ levels^42^,^43^. Because *L*. *bicolor* VOCs failed to promote root hairs and LR on the NADPH oxidase knockout mutant *rhd2*, O_2_^−.^production appears to contribute to LR and root hair growth stimulation by *L. bicolor*. Our results agree with a growing body of evidence that suggests that O_2_^−.^radicals control root growth through cell division maintenance in the meristematic region of roots^44^,^45^. The identity of trichoblasts, the cells capable of producing root hairs, is defined early in the meristem region of the *Arabidopsis* root^46^. Hence, factors influencing cell division and/or maintenance in this region of the root were recently shown to impact on root hair initiation and development^47^. Our data show an increased O_2_^−^ level in the meristematic region of *L*. *bicolor* VOC-exposed *Arabidopsis* roots while DPI, an effective NADPH oxidase inhibitor, interfered with the growth stimulation of root hairs by *L*. *bicolor* VOCs. Taken together, these data suggest that the activation of O_2_^−.^production by *L*. *bicolor* SQTs is a major event required for root hair growth.

A main goal of our study was to identify the nature of the signalling molecule. Fungi and bacteria emit complex mixtures of VOCs, in which the functions of individual compounds are difficult to disentangle^6^,^48^. Recent studies revealed strong differences in the VOC patterns of some ectomycorrhizal fungal species^13^. Strikingly, SQTs were absent in the odours of *C. geophilum* (ascomycota) compared with those of *L. bicolor* or *Paxillus involutus* (basidiomycota)^13^. In contrast to *L. bicolor*^49^, the *C. geophilum* genome does not contain typical sequences for SQT synthases (http://genome.jgi-psf.org/Cenge3/Cenge3.home.html). Here we show that only VOCs from *L. bicolor* and not those emitted by *C. geophilum* induce LR development. As the young healthy *Arabidopsis* seedlings used for this study do not produce SQTs, a role of fungal SQTs as signals for LR stimulation is strongly supported.

To support the hypothesis that SQTs might be causal for LR stimulation, we supplemented *L. bicolor* with lovastatin, which effectively suppressed fungal SQT formation and consequently significantly diminished LR stimulation. Because neither fungal growth nor the synthesis of MTs and OVOCs was affected under these conditions, we concluded that SQTs were the likely regulators of LR development.

All SQTs inhibited by lovastatin are candidate signalling molecules for LR formation. To identify the bioactive compounds, we tested the impact of (–)-thujopsene and β-caryophyllene on root development, because these VOCs were inhibited by lovastatin in the separately grown cultures of *L. bicolor* and were also absent in the headspace of *Arabidopsis* grown in bi-compartmented Petri dishes supplemented with lovastatin in the neighbouring compartment. β-Caryophyllene was chosen, as it was previously found to have several biological functions^10^,^50^ and was furthermore recently implicated as the belowground signalling molecule that stimulates shoot growth and biomass in lettuce plants exposed to *Fusarium oxysporum* in consortium with bacteria^11^. In our system, however, β-caryophyllene was inactive as a growth-promoting substance, underlining the specificity of the SQT-mediated signals within and between various species.

Here, we discovered that (–)-thujopsene, a low-abundant SQT in the emission profile of *L. bicolor*, was sufficient to stimulate LR formation in both plant systems in the absence of the fungus or when the SQT biosynthesis of the fungus was inhibited by lovastatin. (–)-Thujopsene was previously identified in Cupressaceae^51^,^52^, but it has also been detected in the volatile profiles of various fungal clades^50^,^53^,^54^. It is a potent antibiotic compound^50^,^53^,^54^. Its function as a signalling compound in root development, demonstrated here, is novel.

Although we identified (–)-thujopsene as a new signalling molecule, our analysis does not exclude that some of the other, yet untested, candidate SQTs may also stimulate LRs. Future studies, for example, RNA interference suppression of SQT synthases in *L. bicolor* will elucidate whether further SQTs are bioactive molecules that trigger additional LR formation. However, this approach is not straightforward, because SQTs are known as multi-product enzymes^55^ and the genome of *L. bicolor* contains a minimum of eight SQT synthase genes^56^. Furthermore, the carbon source and fungal age may affect the quality and quantity of the fungal volatile profiles^57^,^58^,^59^, leading to variation in the signalling molecules. Previously, VOC emissions were proposed to change the pre-mycorrhization state of *Tuber borchii* Vittad. and *Tilia americana* L. when these interacting partners come into aerial contact with each other^60^. Furthermore, it is possible that the signalling SQTs must interact with plant compounds to trigger LR proliferation. Nevertheless, the identification of a bioactive and another inactive volatile compound stresses that not all SQTs act as LR-stimulating agents, and that host response specificity to these chemicals exists.

A further important question is whether SQT signalling has any potential ecological benefits? Host recognition and colonization are of fundamental importance for ectomycorrhizal fungi to gain access to plant-derived sugars for their own benefit. However, *L. bicolor* SQT signalling also promotes the LR development of non-host plants, and the non-SQT-emitting fungus *C. geophilum* is an abundant mycorrhizal species^61^. These findings indicate that the perception and transduction of SQT signalling is independent of the symbiosis *per se*, and raise questions about the ecological functions of SQTs in plant–fungus communication.

Generally, SQTs are thought to be good candidates for belowground signalling. Hiltpold and Turlings^16^ tested the diffusivity of VOCs in soil and demonstrated that SQTs were the best diffusing compounds in this complex environment. In good agreement with our results, mycorrhizas formed with *C. geophilum* in their natural environment in the forest are hardly ramified, whereas those formed with *Laccaria* species are branched (www.deemy.de) supporting the differential effects of these mycorrhizal species on root development. As mycorrhizal species compete for colonization of the roots tips, where they can tap the plant carbohydrate resources, induction of LR proliferation may constitute an advantage for SQT-emitting species under suitable conditions. Our results are, therefore, an important cornerstone for further ecological studies that address the host range and resource competition of soil microbes.

Another emerging function of fungal VOCs is their growth-promoting effect on plants^13^,^62^. For example, VOCs released by *Trichoderma viride* increased *Arabidopsis* biomass in the absence of direct physical contact between the plant and the fungus^63^. *T. viride* is a widespread, saprotrophic, commensualistic soil fungus, which exerts beneficial effects on plant performance, such as increased adventitious root formation, increased health and growth^63^,^64^. The chemical nature of the growth-inducing volatile(s) was not elucidated^65^. However, it is interesting to note that *T. viride* had the strongest SQT production among a range of different fungal species^13^. Plant growth-promoting effects were also demonstrated in *Nicotiana attenuata* after exposure to mixtures of isolated compounds from the VOC blend of the soil pathogenic fungus *Phoma* sp. GS8-3 (ref. 12), although SQTs were absent in those mixtures. Root proliferation and enhanced growth are also observed in response to VOCs of plant growth-promoting rhizosphere bacteria^65^.

The SQT-induced LR formation shown here may provide nutritional benefits to root-associated fungi, because the root tips exude carbohydrates and amino acids^61^. Concurrently, the enhanced root surface increases the plant potential for mineral and water uptake. Thus, root proliferation, which is most likely beneficial to the fungus and the plant itself, may initiate a feedback mechanism that eventually stimulates growth and increases host fitness^66^. The discovery that LR stimulation can be induced by a single SQT, (–)-thujopsene, is important because it opens new avenues for biotechnological applications of VOCs.

## Methods

### Growth conditions for plants and ectomycorrhizal fungi

Grey poplars (*Populus × canescens* (Aiton) Sm. *Populus tremula* × *Populus alba*; INRA clone 717-1-B4) were multiplied and grown in Petri dish systems with half-strength MS medium solidified with Gerite as reported by Müller *et al.*^67^ or under the conditions reported by Felten *et al.*^3^ *Arabidopsis* (*Arabidopsis thaliana* ecotype Columbia) wildtype and *rhd2* (ref. 19) seeds were surface sterilized and placed on solid *Arabidopsis* medium (AM) (2.3 g l^−1^ MS salt (Duchefa Biochemie B.V., Haarlem, The Netherlands), 1% (w/v) sucrose, 3% (w/v) Gelrite (Carl Roth GmbH & Co. KG, Karlsruhe, Germany) and 0.1% (w/v) 2-(*N*-morpholino)ethanesulfonic acid (MES) sodium salt (Sigma, Steinheim, Germany), pH 6.0 adjusted with HCl. To compare WT growth versus *rhd2*, pH 5.0 was used as root hair development is suppressed in *rhd2* at this pH^19^. After stratification for 2 days at 4 °C in the dark, the seeds were germinated under a 16-h photoperiod at 21 °C.

The ectomycorrhizal fungal strains *Laccaria bicolor* S238N (Maire P.D. Orton) and *Cenococcum geophilum* (Fries) were maintained at 25 °C in permanent darkness on modified Pachlewski medium containing (per 1 l) 0.5 g (w/v) di-ammonium tartrate (Merck, Darmstadt, Germany), 1 g (w/v) potassium dihydrogen phosphate (Merck), 0.5 g (w/v) magnesium sulphate (Duchefa Biochemie B.V.), 5 g (w/v) maltose (Merck), 20 g (w/v) glucose (Carl Roth GmbH & Co. KG), 1 ml of 1/10 diluted Kanieltra microelement solution (Yara, Nanterre, France), 1 ml of 0.1% thiamine-hydrochloride (Merck) and 20 g (w/v) Gelrite (Duchefa Biochemie B.V.), pH 5.5. Three fungal plugs, 8 mm in diameter, were transferred to sterilized cellophane membranes placed in Petri dishes on modified Pachlewski medium P20, as described by Müller *et al.*^67^, and cultured for 10 days at 25 °C in permanent darkness.

### Exposure of poplar explants to fungal VOCs

Poplar plantlets approximately 30 mm tall were placed on the surface of solidified Pachlewski medium P20 (buffered 5 mM MES, pH 5.8) in a bi-compartmented Petri dish (90 mm diameter). A cellophane membrane with the fungal mycelium was either placed directly on the roots (direct contact) or in the adjacent compartment (exposition to fungal VOCs). The Petri dishes were sealed with Parafilm (VWR International GmbH, Darmstadt, Germany) and kept for 10 days in a growth room (21 °C, 16 h light, 134.7 μmol m^−2^ s^−1^ photosynthetically active radiation). The control plants were covered with cellophane and kept under the same growth conditions as the plants with fungal mycelium. The LRs were regularly counted under a Zeiss Stemi SV11 Apo stereomicroscope (Carl Zeiss MicroImaging, Jena, Germany). The experiment was repeated three times with 2–4 plates per treatment.

### Exposure of *Arabidopsis* seedlings to fungal VOCs

To expose *Arabidopsis* seedlings (wildtype and DR5::GFP) to fungal VOCs and to analyse the fungal VOCs profiles, we used glass Petri dishes (diameter 90 mm) separated into two compartments by a Teflon strip^67^. One of the compartments was filled with 10 ml of AM, and the other was filled with 10 ml of modified P20 medium. In the *in vitro* co-culture systems, *Arabidopsis* (WT, DR5::GFP and *rhd2*), *L. bicolor* and *C. geophilum* were either grown alone or in plant–fungus combinations, but always without physical contact between them. For this purpose, 5-day-old *Arabidopsis* seedlings and 10-day-old *L. bicolor* or *C geophilium* mycelium on cellophane membranes were transferred in the respective combinations to the compartmented Petri dishes; *Arabidopsis* was always placed in the AM compartment and the fungi on the membrane in the P20 compartment. The control plates were equipped with a cellophane membrane without fungal mycelium in the P20 compartment. The plates were placed vertically and kept under long day conditions (100 μE m^−2^ s^−1^ light intensity for 16 h light, 8 h darkness) at 21 °C. Ten plates were analysed for each treatment. The number of LRs was counted on days 0, 4, 7 and 10 of cultivation.

The DR5::GFP signal was recorded using an AZ-C1 Macro Laser Confocal Microscope (Nikon GmbH, Düsseldorf, Germany). GFP was excited using the 488-nm laser line in conjunction with a 505–530-nm band-pass filter. Nikon EZ-C1 software was used to quantify the GFP signal. Eight roots per treatment were analysed.

Fungal VOCs were collected after 10 days. To measure the background VOC emissions, 10 plates containing the media and the cellophane membrane without fungal colonies were kept under the same growth conditions.

### Lovastatin treatment

Lovastatin (97%; Alfa Aesar GmbH & Co KG, Karlsuhe, Germany), a specific inhibitor of the mevalonate pathway^68^, was dissolved in ethanol (10 mg ml^−1^), sterile-filtered and added to the P20 media at a final concentration of 2 μg ml^−1^. The plants and *L. bicolor* were cultivated in the bi-compartment system as described above, *Arabidopsis* without and *L. bicolor* with lovastatin. LR formation was measured regularly for 10 days.

### Inhibition of plasmalemma NADPH oxidase by DPI treatment

DPI (Sigma-Aldrich, Deisenhofen, Germany), an inhibitor of NADPH oxidase, was dissolved in dimethylsulfoxide (Carl Roth GmbH & Co. KG) (200 μM) and added into the two-compartmented plates to AM prior to Gelrite solidification at a final concentration of 80 or 100 nM. The other compartment was filled with P20 medium. Five-day-old *Arabidopsis* seedlings were transferred to the compartment containing either AM (control) or AM supplemented with DPI. In the adjacent compartment, 10-day-old *L*. *bicolor* was added as described above. The root hair length and LR number were quantified after 5 days of co-cultivation.

### Detection of superoxide anion radicals by NBT staining

The superoxide anion radicals were detected using the NBT salt (Carl Roth GmbH & Co. KG) assay. NBT was prepared freshly in 50 mM phosphate buffer (pH 6.0) and diluted in liquid AM to a final concentration of 0.5 mM. Seedlings grown for 4 days in the presence or absence of *L. bicolor* VOCs were transferred to AM NBT-containing solution, vacuum infiltrated for 30 s and incubated for 60 min on a shaker at 250 r.p.m. at room temperature in the dark. After incubation, the NBT was removed by two washes (5 min each) with NBT-free liquid AM, the plants were then dehydrated in 100% ethanol for 15 min for tissue clearing, rehydrated in decreasing concentrations of ethanol (80, 60, 40, 20 and 5%, each step 15 min) and finally stored in water. The seedlings were mounted on microscope slides in 20% chloral hydrate in 50% glycerol and visualized using a Zeiss Axiovert 200M MOT (Carl Zeiss MicroImaging) microscope equipped with differential interference contrast (DIC) optics.

### Exposure of *Arabidopsis* and poplar to individual VOCs

*Arabidopsis* seedlings were grown on AM and poplar plantlets on P20 medium in one half of the bi-compartmented Petri dishes, as described above. The adjacent compartment was equipped with sterile filter paper (1 × 1 cm; Whatman, Maidstone, UK). (–)-Thujopsene (≥97%; Sigma) and β-caryophyllene (≥80%; Sigma) were diluted to final concentrations of 1, 10, 100 and 1,000 p.p.b. in *n*-pentane (HPLC grade, Carl Roth GmbH & Co. KG). Thirty microlitres of the solution was dropped on the filter paper. The control plates were equipped with 30 μl of *n*-pentane. The treatment was performed once at the beginning of the experiment. The Petri dishes were immediately sealed with Parafilm and the plants were grown under controlled environmental conditions as described before. The LRs were quantified after 0, 4, 7 and 10 days of treatment. VOCs were collected for 6 h after 3, 7 and 10 days. Because no significant changes of the VOCs patterns were observed, only data for 10 d.h.c.c. are shown. Furthermore, *Arabidopsis* seedlings or poplar plantlets were grown in bi-compartmented plates with or without *L. bicolor* with lovastatin, as described above. The compartment with the inhibited fungus was supplemented with 30 μl of 100 p.p.b. (–)-thujopsene solution or *n*-pentane as the control. The LRs were quantified after 10 days (*n*=5–10 plates per treatment).

### Test of CO_2_ effects on LR formation

CO_2_-trapping experiments were performed in square Petri dishes (12 × 12 cm, Carl Roth GmbH & Co. KG), which were used as the compartment to enclose the bi-compartmented plates with plant and fungus and the Ba(OH)_2_ solution for CO_2_ removal (Supplementary Fig. 1a). For this purpose, two 6-ml-flasks (VWR International GmbH) containing 5 ml 0.1 M Ba(OH)_2_ solution (Carl Roth GmbH & Co. KG) were placed in each Petri dish after ref. 14. To increase the surface for trapping CO_2_, an 11 × 1-cm filter paper (Whatman) was inserted into each flask. *Arabidopsis* and *L. bicolor* or *C. geophilum* were cultivated in the bi-compartmented plate (as described above), which was placed without a lid in the middle of the square Petri dish. The square Petri dishes were closed with their lids and sealed with Parafilm. Five plates were analysed for each treatment. The number of LRs was quantified after 0 and 10 days of treatment. The dry weight of precipitated BaCO_3_ was determined by filtering the solution and drying the filter paper for 4 days at 50 °C as described in ref. 14.

Because sealing the experimental set-up with Parafilm can have an effect on plant growth^14^, the co-culture of *Arabidopsis* with *L. bicolor* or *C. geophilum* was repeated in Petri dishes on which the lid was placed without sealing with Parafilm.

### Sampling and analysis of fungal VOCs

VOCs were collected in the headspace from fungi, *Arabidopsis* and their combinations after 10 days. Ten replicates of each plate with the fungus, *Arabidopsis* or both were studied. Control plates without plants or mycelium were sampled for background correction. The VOCs were collected for 6 h from sealed Petri dishes by headspace sorptive extraction using the stir bar sorptive extraction method with Gerstel Twisters (Gerstel GmbH & Co. KG, Mülheim an der Ruhr, Germany)^69^, as described in detail^13^. Briefly, twisters (10 mm length) were coated stir bars with a 0.5-mm-thick film of polydimethylsiloxane, which passively absorbs VOCs. The samples were desorbed with a temperature gradient, refocused with TENAX and separated by capillary gas chromatography using a thermo-desorption unit (Gerstel GmbH & Co) coupled to a gas chromatograph–mass spectrometer (GC–MS; GC model: 7890A; MS model: 5975C; Agilent Technologies, Santa Clara, CA, USA). Compounds were separated with a capillary GC column ((14%-cyanopropyl-phenyl)-methylpolysiloxane; 70 m × 250 μm, film thickness 0.25 μm; Agilent J&W 122-5562G, DB-5MS+10 m DG). The carrier gas was helium, with a constant flow rate of 1.2 ml min^−1^. Further technical and chromatographic conditions have been described in detail^15^.

The chromatograms were analysed by the enhanced ChemStation software (MSD ChemStation E.02.01.1177, 1989–2010 Agilent Technologies), as described earlier^13^. The VOCs previously reported in the literature for *L. bicolor*, *C. geophilum*^15^,^21^ and *A. thaliana*^70^ were manually screened in the total ion counts (TICs) of all chromatograms. Compounds with an identification quality above 70% in the Wiley data library and that were present in more than three samples were checked for their *m/z* ratio. Only compounds with an *m/z* ratio that matched those of the Wiley data library were retained in the final data set. In total, 43 compounds were removed from the data set, because they were already present in the background odour of the plates and experimental set-up (Supplementary Table 2).

The TIC of each VOC in the final data set was recalculated from the absolute abundance of the first representative *m/z* to eliminate noise. For quantification, the following external standards were used: linalool for oxygenated MTs, trans-caryophyllene for SQTs and nerolidol for oxygenated SQTs. OVOCs were quantified relative to toluene, which was used as the external standard. For the comparison of fungal VOC profiles, the TICs were normalized to the fungal mycelium area. The plates were scanned and the mycelium area was measured using ImageJ software (http://rsbweb.nih.gov/ij/download.html). In addition, plates with 30 μl of 100 p.p.b. (–)-thujopsene in *n*-pentane and *n*-pentane were analysed for 6 h.

### Statistical analysis

Statistical analyses were conducted with R (R Core Team 2012) and Graphpad prism (GraphPad Software, San Diego, CA). Data violating the assumption of normality were log-transformed before statistical analysis. Mean values were compared by the Student’s *t*-test or analysis of variance, followed by the *post hoc* Tukey’s Honest Significant Difference test. A generalized linear model with quasi-Poisson distribution was fitted and analysed by analysis of deviance. *P* values ≤0.05 indicated significant differences.

## Author contributions

A.M., A.P., F.A.D., J.F., J.-P.S., M.R. and K.P. designed the experiments. A.M., F.A.D., J.F., H.L. and M.M.v.D. conducted poplar and *Arabidopsis* root assays with *L. bicolor*. F.A.D. and H.L. conducted auxin and ROS assays. A.M. and M.R. analysed volatiles. A.M. and M.M.v.D. conducted thujopsene bio-assays. A.M., A.P., F.A.D., J.F., J.-P.S., M.R., K.P. and V.L. analysed data, and A.M., A.P., F.A.D., J.-P.S., M.R. and K.P. wrote the manuscript. All authors commented on the final version.

## Additional information

**How to cite this article:** Ditengou, F. A. *et al.* Volatile signalling by sesquiterpenes from ectomycorrhizal fungi reprogrammes root architecture. *Nat. Commun.* 6:6279 doi: 10.1038/ncomms7279 (2015).

## Supplementary Material

Supplementary Information Supplementary Figures 1-3, Supplementary Tables 1-2 and Supplementary References

## Figures and Tables

**Table 1:** Concentrations of volatile organic compounds (VOCs) in separate cultures and co-cultures of *Arabidopsis* and mycorrhizal fungi.

**Group**	**Compound no.**	**Id. no**	**CAS registry**	**Concentration (pmol)**							
				**At**	**Lb**	**At+Lb**	**Lb+Lov**	**At+Lb+Lov**	**At+Lov**	**Cg**	**At+Cg**
MT	3-Carene	1	13466-78-9	3.5±2.7	6.0±4.3	4.0±2.1	4.9±2.9	3.2±1.7	4.2±3.0	4.3±3.2	ND
SQTs	α-Ylangene	2	14912-44-8	ND	1.7±1.7	2.2±1.7	2.1±1.4	ND	ND	ND	ND
	β-Elemene	3	515-13-9	ND	7.5±2.6	2.1±1.2	3.3±1.4	0.6±0.4	ND	ND	ND
	β-Caryophyllene	4	87-44-5	ND	8.5±4.0	2.1±1.1	ND	ND	ND	ND	ND
	(−)-Thujopsene	5	470-40-6	ND	1.9±1.8	0.4±0.4	ND	ND	ND	ND	ND
	β-Selinene	6	17066-67-0	ND	7.2±4.0	0.4±0.4	1.8±1.5	0.4±0.4	ND	ND	ND
	α-Amorphene	7	23515-88-0	ND	1.1±1.1	3.4±1.8	2.9±1.5	0.4±0.4	ND	ND	ND
	γ-Cadinene	8	39029-41-9	ND	4.7±4.7	ND	ND	0.3±0.3	ND	ND	ND
	γ-Selinene	9	515-17-3	ND	15.8±4.2	2.6±1.7	7.6±3.7	2.5±1.1	ND	ND	ND
	α-Muurolene	10	31983-22-9	ND	63.6±13.3	50.9±14.3	19.9±8.3	11.2±3	ND	ND	ND
	δ-Cadinene	11	483-76-1	ND	10.0±2.1	6.0±1.6	4.1±1.4	2.0±0.5	ND	ND	ND
	(−)-Isoledene	12	95910-36-4	ND	1.0±1.0	1.1±0.8	ND	0.4±0.4	ND	ND	ND
	Epizonarene	13	41702-63-0	ND	6.3±2.2	1.3±1.0	2.2±1.4	1.2±0.4	ND	ND	ND
	Cadina-1,4-diene	14	29837-12-5	ND	0.1±0.1	ND	ND	ND	ND	ND	ND
	Valencene	15	4630-07-03	ND	ND	0.4±0.4	ND	ND	ND	ND	ND
OVOCs	2-Penta 4,4-di-methyl-none, Heptadecane	16	590-50-1	ND	43.0±18.2	2.3±2.3	ND	ND	6.4±6.4	ND	ND
		17	629-78-7	90.6±75.2	ND	2.7±2.7	10.9±10.9	18.2±9.3	82.2±20.4	ND	ND

MT, monoterpenes; ND, not detected; OVOCs, other volatile organic compounds; SQT, sesquiterpene.

The concentrations of MT, SQTs and OVOCs emitted by *Arabidopsis* seedlings (At), *L. bicolor* mycelium (Lb), *C. geophilum* mycelium (Cg) and *Arabidopsis* grown in bi-compartmented plates in the presence of *L. bicolor* or *C. geophilum* without those compounds, whose origin could not be unequivocally attributed to living organisms (Supplementary Table 2). Lovastatin (Lov), an inhibitor of sesquiterpene biosynthesis, was added to the fungal medium at a concentration of 5 μM. The data indicate mean values (*n*=10, ±s.e.).
