# Supplementary material for: Volatile signalling by sesquiterpenes from ectomycorrhizal fungi reprogrammes root architecture
Source: Nat Commun. 2015 Feb 23;6:6279. doi: 10.1038/ncomms7279 (PMC4346619; doi:10.1038/ncomms7279)
Supplement: Supplementary Information — Supplementary Figures 1-3, Supplementary Tables 1-2 and Supplementary References [file ncomms7279-s1.pdf]

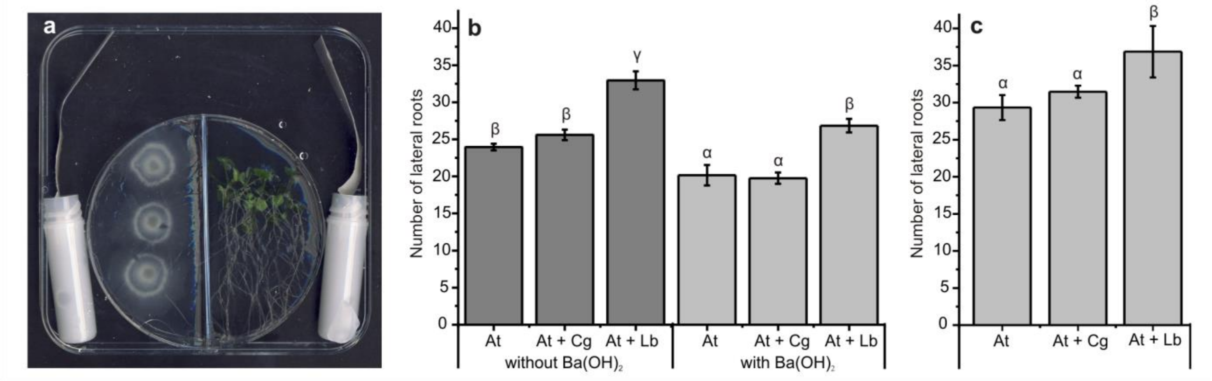

Supplementary Figure 1 | **Stimulation of lateral root (LR) development in *Arabidopsis* by *L. bicolor* is not due to fungal CO_2.**

(**a**) *Arabidopsis* seedlings grown in the presence of *L. bicolor* in a CO_2 trapping system with $Ba(OH)_2$. Square Petri dishes were equipped with a bi-compartmented plate containing fungus and plant. Two flasks with 5 ml 0.1M $Ba(OH)_2$ solution were placed in each square Petri dish. To increase the surface for trapping CO_2 a filter paper was inserted into each flask. **(b)** LR development of *Arabidopsis* (At) was significantly stimulated in the presence of *L. bicolor* (Lb) and not in the presence of *C. geophilum* (Cg) with or without trapping of CO_2 with $Ba(OH)_2$. The presence of $Ba(OH)_2$ diminished LR formation, but did not abolish the Lb effect. Different letters indicate significant differences at $P < 0.05$ (Tukey-test); data are means ± SE (n = 5 plates per treatment, 5 seedlings per plate). After 10 dhcc the dry mass of $BaCO_3$ in all plant fungal combinations was 17.2 ± 1.0 mg and showed no significant differences. (c) *L. bicolor* (Lb) VOCs, but not *Cenococcum geophilum* (Cg) VOCs stimulate LR development in *A. thaliana* seedlings (At) in bi-compartmented Petri dishes, which were not sealed with Parafilm to facilitate CO_2 exchange. Different letters indicate significant differences between the treatments (Tukey-test, P <0.05; n = 3; mean ± SE). Note, that the formation of LR in non-sealed plates was greater than in sealed plates (Supplementary Fig. 1b).

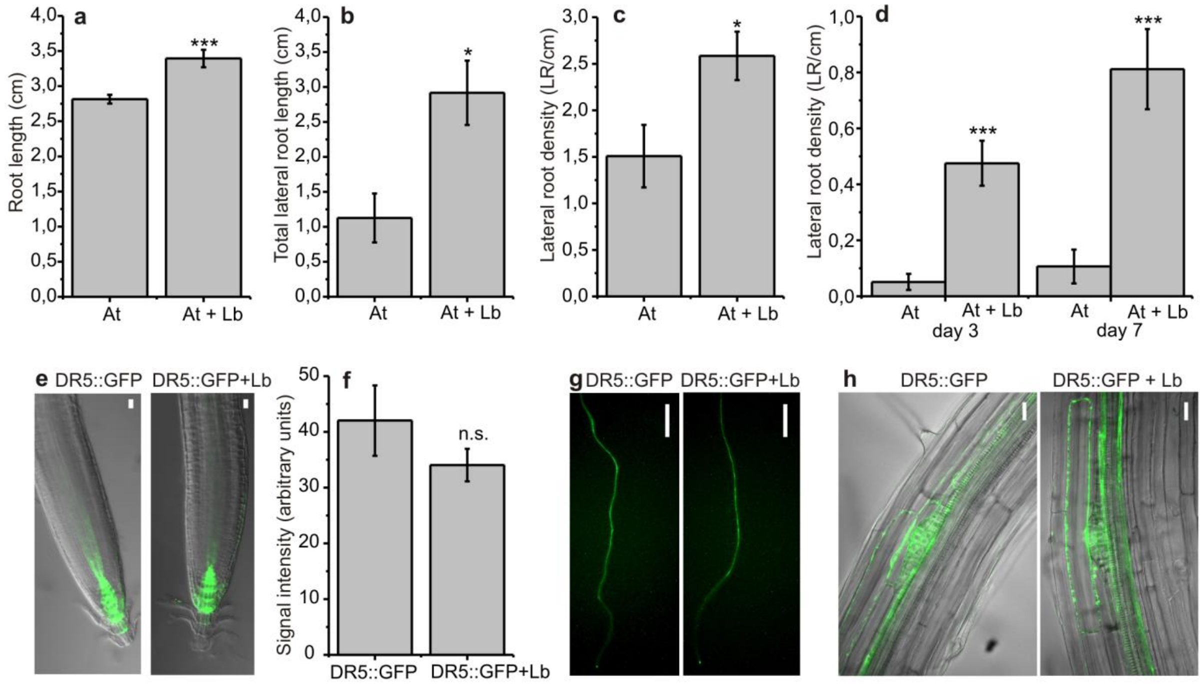

Supplementary Figure 2│ **Impact of *Laccaria bicolor* volatile organic compounds (VOCs) on lateral root (LR) initiation of *Arabidopsis thaliana*.**

(a) *L. bicolor* VOCs promote main root growth (n = 31, mean ± SE, *** Student´s T test *P* < 0.001). (**b**) *L. bicolor* promotes the elongation of LRs. **(c)** *L. bicolor* VOCs increase LR density (n = 28, mean ± SE, * Student´s T *P* < 0.05). (**d**) *L. bicolor* VOCs increase LR initiation density (n= 30, mean ± SE, Student´s T test P < 0.001). **(e-g)** Root tips and LRs of *Arabidopsis thaliana* expressing green fluorescent protein (GFP) under the auxin responsive DRE5 promoter do not show increased fluorescence in the presence of *L. bicolor*. **(e)** Root tips. **(f)** Quantification of the GFP signal from roots as presented in (e), n = 10, mean ± SE, Student´s T test: P = n.s = not significant **(g)** Entire root grown after transfer. Scale bar: 1000 µm **(h)** LRs. Scale bar: 20µm.

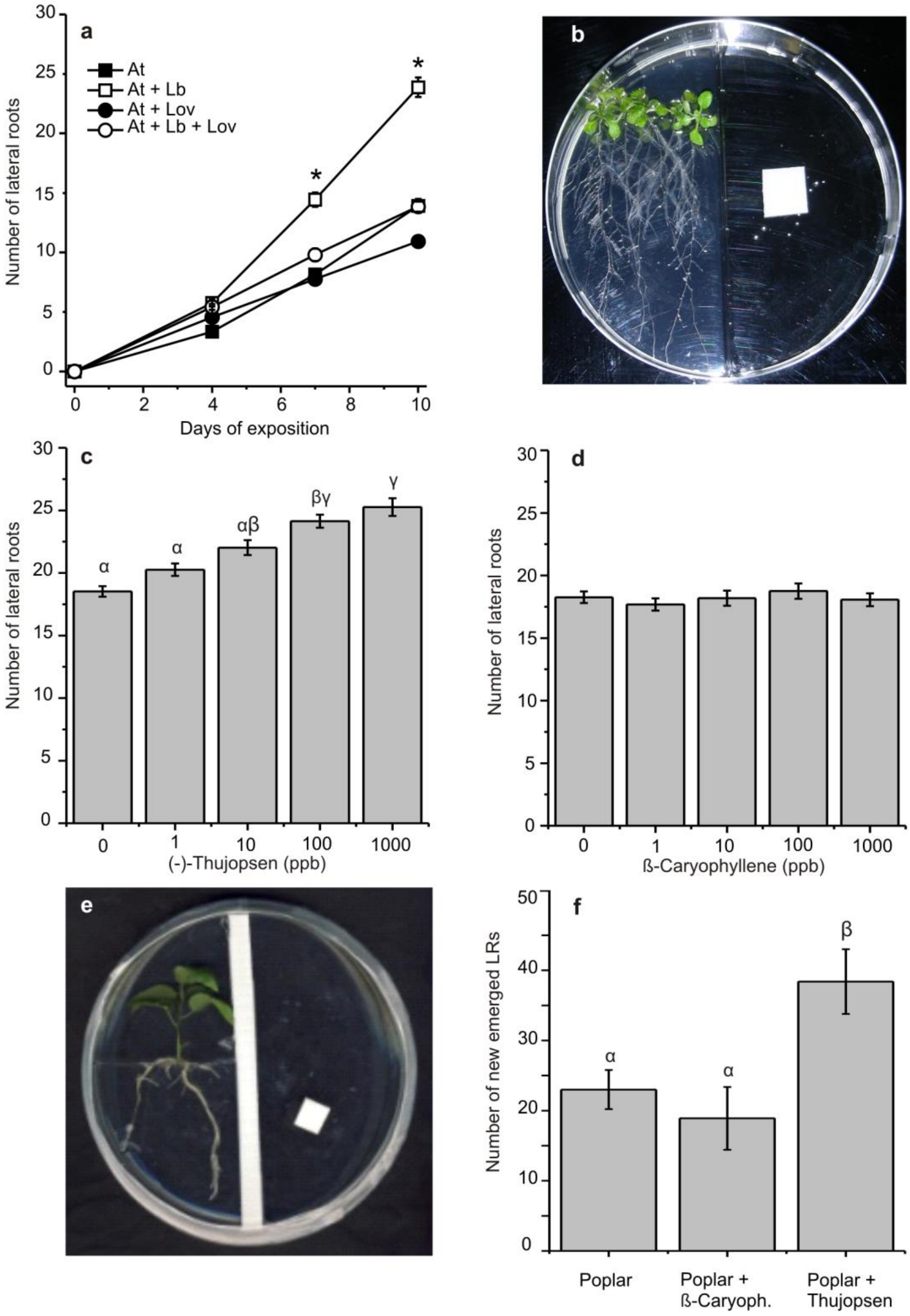

Supplementary Figure 3 │ **Response of lateral root (LR) formation of *Arabidopsis thaliana* and *Populus* x *canescens* to increasing concentrations of sesquiterpenes.**

(a) Lateral root formation of *Arabidopsis* seedlings in a bi-compartmented Petri dish without physical contact to the second compartment, which contained fungal medium (At), lovastin (Lov, 5µM), L. bicolor (Lb) or Lb+Lov. Data indicate means ± SE (n = 10). Stars indicate significant differences at P < 0.05 (t test). When error bars are not visible, they are smaller than the symbols. **(b)** *Arabidopsis* seedlings exposed to a filter paper with 30 µl of 100 ppb (-)-thujopsen in n-pentane in a bi-compartmented Petri dish. **(c)** (-)-Thujopsen significantly stimulated lateral root formation of *Arabidopsis thaliana* at concentrations of 100 ppb and above. Controls were exposed to n-pentane. **(d)** ß-Carophyllene did not induce LR formation in *Arabidopsis thaliana*. Data in (c) and (d) indicate means ± SE (n = 10 plates, each with 5 seedlings per treatment). Different letter indicate significant differences at P < 0.05 (one-way analysis of variance) **(e)** Poplar plants exposed to a filter paper with 30 µl of 100 ppb (-)-thujopsen in n-pentane in a bi-compartmented Petri dish. **(f)** (-)-Thujopsen (100 ppb), but not ß-caryophyllene (100 ppb) induced LR formation in *Populus* x *canescens*. Data indicate means ± SE (n = 5 plates, each with one plant per treatment). Different letters indicate significant differences at $P < 0.05$ between the treatments (HSD test).

Supplementary Table 1 | **Identification of the volatile organic compounds collected in the headspace of separate and co-cultures of *Arabidopsis thaliana* and the mycorrhizal fungi *L. bicolor* and *C. geophilum*.**

Compound	Id. no.	CAS registry number	RT (min)	*I*	1st m/z	Absolute abundance (%) in TIC of 1st m/z	2nd m/z	3rd m/z
Monoterpenes								
3-Carene	1	13466-78-9	18.60	999	93	10.2	77	136
Sesquiterpenes								
α-Ylangene	2	14912-44-8	34.63	1389	105	5.7	119	204
β-Elemene	3	515-13-9	35.32	1406	93	5.5	81	107
β-Caryophyllene	4	87-44-5	36.52	1435	93	1.6	69	133
(-)-Thujopsene	5	470-40-6	38.21	1476	119	6.0	105	121
β-Selinene	6	17066-67-0	38.30	1478	189	10.6	133	204
α-Amorphene	7	23515-88-0	38.55	1484	161	17.9	93	119
γ-Cadinene	8	39029-41-9	38.55	1484	161	4.0	41	91
γ-Selinene	9	515-17-3	38.60	1485	189	9.3	133	204
α-Muurolene	10	31983-22-9	39.07	1497	105	11.0	161	204
δ-Cadinene	11	483-76-1	39.86	1516	161	13.8	119	204
(-)-Isoledene	12	95910-36-4	39.97	1519	161	8.8	105	91
Epizonaren	13	41702-63-0	40.11	1522	161	9.9	204	81
Cadina-1,4-diene	14	29837-12-5	40.35	1528	119	14.8	105	161
Valencene	15	4630-07-3	40.45	1530	161	3.5	93	79
Other VOCs								
2-Pentanone,4,4-dimethyl-	16	590-50-1	9.81	684	43	31.0	57	114
Heptadecane	17	629-78-7	43.49	1604	57	9.5	43	71

CAS: Chemical Abstract Service
I: Retention index calculated according to van Den Dool and Kratz[1]
m/z: Mass to charge ratio

Supplementary Table 2 | **Volatile organic compounds detected in the headspace of the experimental set up and potential contaminations.**

Compound	CAS registry number	RT (min)	*I*
Chloroform	67-66-3	7.06	608
Hexamethylcyclotrisiloxane	541-05-9	10.54	803
Nonane	111-84-2	13.68	880
α-Pinene	80-56-8	15.22	917
1-Decene	872-05-9	17.34	969
Decane	124-18-5	17.95	984
Heptane,2,2,6,6-tetramethyl-4-methylene-	141-70-8	17.98	984
Isopropyl palmitate	141-70-8	17.98	984
Octanal	124-13-0	18.20	990
Trans-β-ocimene	3779-61-1	18.53	998
3-Heptene,2,2,4,6,6pentamethyl	123-48-8	19.16	1013
Undecane	1120-21-4	22.56	1096
1,1,3,3-Tetramethylindane	4834-33-7	25.98	1179
Dodecane	112-40-3	27.08	1205
Decanal	112-31-2	27.42	1214
Tridecane	629-50-5	31.34	1309
2,6,10-Trimethyldodecane	3891-98-3	34.38	1383
Tetradecane	629-59-4	35.37	1407
3-Octadecene,(e)	7206-19-1	38.57	1485
Pentadecane	629-62-9	39.07	1497
2,6-bis(1,1Dimethylethyl)-4-methylphenol	128-37-0	39.24	1501
Phenol,2,5bis(1,1dimethylethyl)	5875-45-6	39.37	1504
Hexadecane	544-76-3	41.61	1559
3(E)-3-Eicosene	74685-33-9	42.77	1587
1-Pentadecene	13360-61-7	44.58	1631
Isolantolactonoidbutenolidea	NA	44.66	1633
Octadecane	593-45-3	44.91	1639
1-Octadecene	112-88-9	44.99	1641
Isopropylmyristate	110-27-0	45.22	1646
5-Butyl-6-hexyl-2,3,3a,4,5,6,7,7a-octahydro-1H-indene	55044-36-5	45.32	1649
Neophytadiene	504-96-1	45.38	1650
Diisobutyl phthalate	84-69-5	45.75	1659
1-Heptadecene	6765-39-5	46.06	1667
1-Tetradecene	1120-36-1	46.09	1667
1-Hexadecene	629-73-2	46.11	1668
Cyclohexadecane	295-65-8	46.29	1672
Cyclotetradecane	295-17-0	46.30	1673
1-Nonadecene	18435-45-5	46.32	1673
1-(1,5-Dimethylhexyl)-4-(4-methylpentyl)cyclohexane	56009-20-2	46.52	1678
Nonadecane	629-92-5	46.69	1682
14-β-H-Pregna	NA	46.95	1688
1-2-Trimethylsiloxy-1,1-dideuteriovinyl-4-trimethylsiloxybenzene	NA	47.30	1697
Eicosane	112-95-8	48.27	1720

Many of these compounds were also responsive to the presence of mycorrhizal fungi or *Arabidopsis thaliana*, e.g. various halogenated compounds, but were not considered for the purpose of this study as their origin from living organisms could not be unequivocally determined. RT: Retention time, CAS: Chemical Abstract Service, NA = not available
I: Retention index calculated according to van Den Dool and Kratz[1]

Supplementary References

1. Van Den Dool, H. & Kratz, P.D. A generalization of the retention index system including linear temperature programmed gas-liquid partition chromatography. *J. Chromatogr.* **2**, 463-471 (1963).
